# Feasibility, coverage, and inter-rater reliability of the assessment of therapeutic interaction by a humanoid robot providing arm rehabilitation to stroke survivors using the instrument THER-I-ACT

**DOI:** 10.3389/frobt.2023.1091283

**Published:** 2023-02-23

**Authors:** Thomas Platz, Ann Louise Pedersen, Stephanie Bobe

**Affiliations:** ^1^ Neurorehabilitation research group, University Medical Centre, Greifswald, Germany; ^2^ BDH-Klinik Greifswald, Institute for Neurorehabilitation and Evidence-Based Practice, An-Institut, University of Greifswald, Greifswald, Germany

**Keywords:** robot, arm, stroke, interaction, social, reliability, rater, rehabilitation

## Abstract

**Objective:**

The instrument THERapy-related InterACTion (THER-I-ACT) was developed to document therapeutic interactions comprehensively in the human therapist–patient setting. Here, we investigate whether the instrument can also reliably be used to characterise therapeutic interactions when a digital system with a humanoid robot as a therapeutic assistant is used.

**Methods:**

*Participants and therapy:* Seventeen stroke survivors receiving arm rehabilitation (i.e., arm basis training (ABT) for moderate-to-severe arm paresis [n = 9] or arm ability training (AAT) for mild arm paresis [n = 8]) using the digital therapy system E-BRAiN over a course of nine sessions. *Analysis of the therapeutic interaction:* A total of 34 therapy sessions were videotaped. All therapeutic interactions provided by the humanoid robot during the first and the last (9th) session of daily training were documented both in terms of their frequency and time used for that type of interaction using THER-I-ACT. Any additional therapeutic interaction spontaneously given by the supervising staff or a human helper providing physical assistance (ABT only) was also documented. All ratings were performed by two trained independent raters.

**Statistical analyses:**

Intraclass correlation coefficients (ICCs) were calculated for the frequency of occurrence and time used for each category of interaction observed.

**Results:**

Therapeutic interactions could comprehensively be documented and were observed across the dimensions provision of information, feedback, and bond-related interactions. ICCs for therapeutic interaction category assessments from 34 therapy sessions by two independent raters were high (ICC ≥0.90) for almost all categories of the therapeutic interaction observed, both for the occurrence frequency and time used for categories of therapeutic interactions, and both for the therapeutic interaction performed by the robot and, even though much less frequently observed, additional spontaneous therapeutic interactions by the supervisory staff and a helper being present. The ICC was similarly high for an overall subjective rating of the concentration and engagement of patients (0.87).

**Conclusion:**

Therapeutic interactions can comprehensively and reliably be documented by trained raters using the instrument THER-I-ACT not only in the traditional patient–therapist setting, as previously shown, but also in a digital therapy setting with a humanoid robot as the therapeutic agent and for more complex therapeutic settings with more than one therapeutic agent being present.

## 1 Introduction

While neuro-disabilities including those that are stroke-related are on the rise globally (Nguyen et al., 2019), neurorehabilitation at the same time offers treatment to combat disabilities (Stroke Units Trialist Collaboration, 2013; Langhorne and Ramachandra, 2020) to a large degree by therapeutic training that promotes functional recovery, i.e., “neural repair therapy” (Joy and Carmichael, 2021), based on mechanisms of brain plasticity (Kane and Ward, 2021). Such restorative therapy frequently implies a prolonged intensive and specific training, led by therapists. Such training therapy intends to achieve goals related to functional recovery that promotes a patient’s capacities for everyday life activities and, consecutively, participation in social life. To achieve such goals, the process of therapy itself, i.e., during therapeutic sessions, needs to be appropriately structured to enable a patient to perform the specifically chosen type of (“neural repair”) training in an engaged and committed way, frequently over extended periods of time (Michael et al., 2020).

As patients are not experts in such trainings, therapists need to provide information, e.g., training specifications and instructions, and provide feedback that matches the focus of the training, e.g., knowledge of the performance, information about how the training task was realised with one’s body or knowledge of the result, the measurable result of the training behaviour such as time and precision. In addition, therapists promote a positive and enduring work alliance by taking interest in the other person, responding to their needs, or, at times, by introducing their own personal experiences and alike. All these activities can be referred to as therapeutic interactions. Together, they can be regarded critical for training therapy to meet its process goals and, hence, are of great interest in rehabilitation research.

For a long time, however, no validated tools were available to comprehensively assess therapeutic interactions until recently, when the instrument THER-I-ACT was specifically constructed to assess a therapeutic interaction (Platz et al., 2021). The types of therapeutic interactions covered by THER-I-ACT include various categories of information provision (e.g., goal-related interactions, training specifications, and instructions), feedback (e.g., knowledge of performance or result and added social stimuli), and bond-related interactions (e.g., showing interest in the other person, responsivity to cues provided by the interaction partner, and solving conflicts). THER-I-ACT promotes a reliable manual-based assessment of these therapeutic interactions both in terms of the frequency of occurrence and time used for such interactions. The frequency of occurrence denotes the number of episodes observed for a specific category of interaction within a therapeutic session; the time used documents the time used for the episodes of a given category of therapeutic interaction.

Social, including therapeutic interaction, is no longer a domain of human–human interaction only, but has recently been introduced to the technology of socially interactive humanoid robots (HRs). Such HRs have a human-like appearance and frequently the capability to move body parts and might be equipped with technical “vision” or “hearing” and, most notably, with a capacity for social interaction. Among the user cases that have been investigated so far are HR companions providing interactions, supporting everyday life, or facilitating cognitive or physical training for the elderly (Andtfolk et al., 2022) or individualised social interactions for long-term care facility residents with dementia (Chen et al., 2020). In addition, HRs were used to improve social skills for children with autism spectrum disorders (Mengoni et al., 2017) or as coaches for physical exercises to promote arm function in children with cerebral palsy (Martin et al., 2020) or stretching exercises for low back pain relief (Blanchard et al., 2022). Furthermore, HRs have been designed and used to assist post-stroke patients in performing exercises during their rehabilitation process, at times for over extended periods of time (Koren et al., 2022), or with applications that were designed to provide human-like comprehensive guidance and interactions during therapeutic sessions (Forbrig et al., 2022). For such training-based therapy, therapeutic interactions, now provided by an HR, is a critical element.

This research was set forth to assess whether therapeutic interactions by an HR could comprehensively and reliably be assessed with the instrument THER-I-ACT that had been developed and validated for the situation when a human therapist interacts with patients therapeutically. In addition, this research intended to extend the scope of the assessment of therapeutic interactions using THER-I-ACT for situations where not only a therapist and a patient are present but also when an HR is the primary therapeutic coach, with the supervising staff (human being) being present at the same time or with the presence of an additional human “helper” who provides physical assistance as needed. The extended research question here was whether therapeutic interactions by either the humanoid robot, supervising staff, or helper when simultaneously present could comprehensively and reliably be assessed using the instrument THER-I-ACT.

## 2 Methods

### 2.1 Participants

The participants for this study could be stroke survivors who participated in the clinical trial E-BRAiN (Evidence-based Robot Assistant in Neurorehabilitation; https://clinicaltrials.gov/ct2/show/NCT05152433) and completed the 2-week course of the humanoid robot-led therapy at one of the two study centres, i.e., the Universitätsmedizin Greifswald or the BDH-Klinik Greifswald. The eligibility criteria for the E-BRAiN trial were as follows: age ≥18 years, history of stroke (ischaemic stroke, non-traumatic intracerebral haemorrhage, and subarachnoidal haemorrhage), either stroke-related upper extremity paresis or visual neglect, not pregnant or breastfeeding, not living in custody, and providing informed consent.

The research was approved by the institution’s review board (Ethikkommission der Universitätsmedizin Greifswald; date of approval: 10.05.2021).

### 2.2 Therapy

Stroke survivors included in this research [n = 17] participated in the clinical trial E-BRAiN and completed the 2-week course of humanoid robot-led therapy. They received ten arm rehabilitation sessions (one introductory session with a human therapist and nine sessions with the humanoid robot) as either the arm basis training (ABT) for moderate-to-severe arm paresis [n = 9] or arm ability training (AAT) for mild arm paresis [n = 8] using the digital therapy system E-BRAiN with a humanoid robot as the therapeutic agent.

Stroke survivors with a residual arm and hand paresis who could move their arm well against gravity (shoulder abduction and elbow flexion strength ≥4 out of 5 strength grades) have no more than moderate paresis of their fingers (index and thumb strength ≥3 out of 5 strength grades), preserved selective movements of their fingers, and were capable of grasping small objects qualified for the category “mild arm paresis”, and hence, in AAT, those with more severe arm paresis (not fulfilling ≥1 criterion for mild arm paresis) fell into the category “moderate-to-severe arm paresis” and received ABT.

The AAT trains the sensorimotor efficiency by repetitive training. Eight tasks address different sensorimotor abilities such as aiming, steadiness, speed of finger movements, and finger and gross manual dexterity. During each therapeutic session, each of the eight tasks is repetitively practiced at the performance limit over four runs, each lasting approximately 1 min, while feedback as a summary of the knowledge of the results is provided intermittently. The trainee aims at improving her/his sensorimotor performance constantly. The ABT trains the selective movement capacity for individual joints of the arm and hand by repetitive movement attempts across the full range of passive movements in various directions for the shoulder, elbow, forearm, wrist, and fingers, addressed individually in a sequential way and physically assisted as needed. The graded exercises start with a single degree of freedom of the movements for all segments of the affected limb; each movement (selective active movement across the full range of a passive movement) is performed repetitively each day with assistance (e.g., weight support and completion of a movement) by a healthy subject (in the conventional setting a trained therapist) as needed (Platz, 2004; Platz et al., 2009).

During the first introductory session, the participants learnt how to perform the standardised training (AAT or ABT), while the human therapist in addition noted and decided on the individualisations indicated that were then used as prescriptions for the digital therapy system E-BRAiN.

During the nine consecutive sessions, the therapeutic training was led by the humanoid robot (“robot”) providing therapeutic interaction as implemented in the digital system based on both training standards and individualisation algorithms. For safety reasons and to step in if needed, all humanoid robot-led sessions were accompanied by a supervising staff (“therapist”). The participants with moderate-to-severe arm paresis receiving the arm basis training could not necessarily perform all training movements by themselves and could perform them only to a variable degree, e.g., only with a limb weight support or over a limited range. Since the robot could not provide physical assistance and served as a social agent only (therapeutic interaction), these participants received physical assistance as needed provided by a “helper.” The helper was not a trained therapist, but was also using the instructions provided by the robot. In this research, the helper was a non-therapeutic staff member (e.g., a person with administrative or scientific duties).

### 2.3 Video recording of sessions and THER-I-ACT ratings

The robot-led training sessions had audio-visually been video recorded twice, both on the first and the last (9th) session. Hence, for each participant, data of the two sessions were available for offline rating of the therapeutic interactions. The videorecorder was placed to cover the therapy scenario, its agents, the interface used for visual displays (tablet or monitor), and to show the training activity currently performed. Since the therapeutic interaction implemented in the system is either verbal or audio-visual accompanied by verbal phrases, the audio-recording was also mandatory and used for the analysis of therapeutic interactions.

**FIGURE 1:**
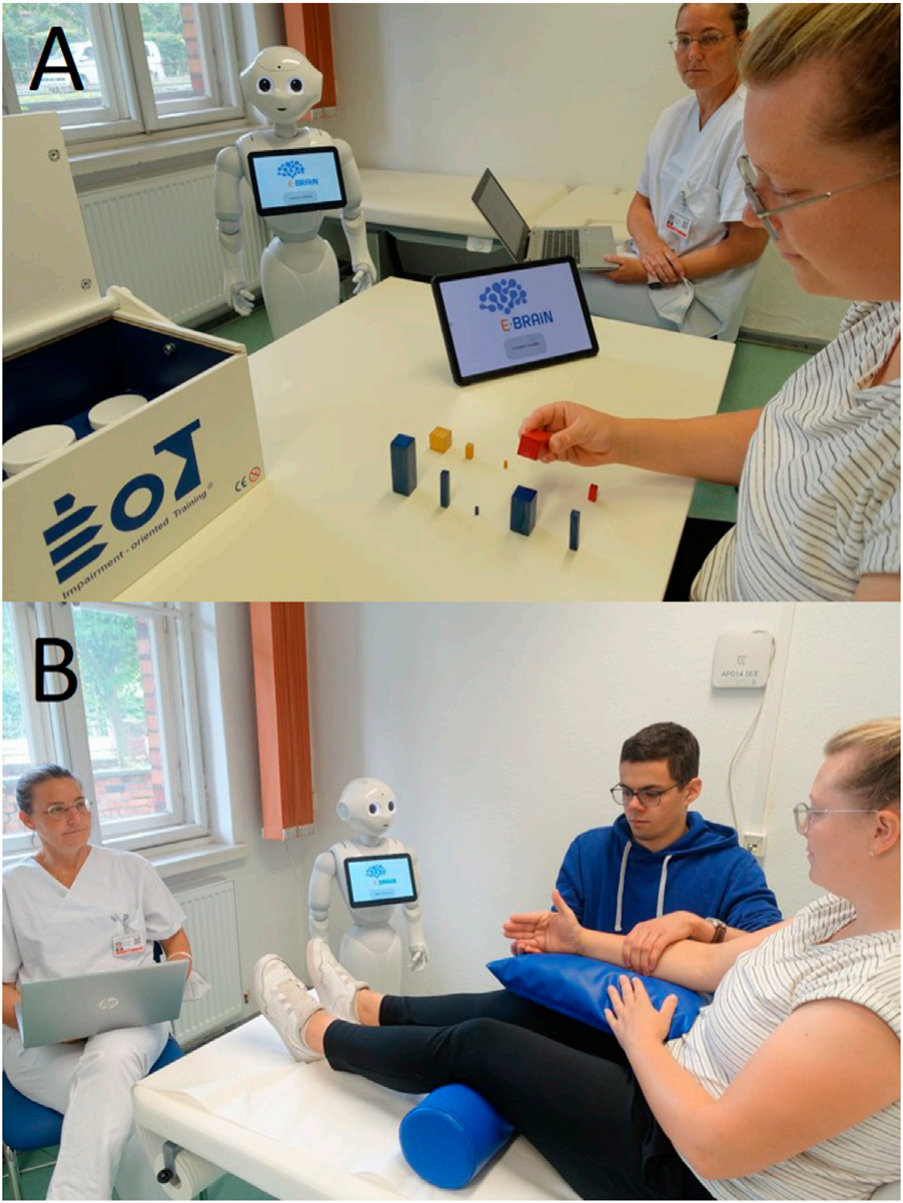
Training setup. **(A)** AAT for stroke patients with mild arm paresis, a scenario with the patient, humanoid robot, and supervising staff. **(B)** ABT for patients with moderate-to-severe arm paresis, a scenario with the patient, humanoid robot, helper, and supervising staff. During nine consecutive sessions over 2 weeks, the therapeutic training (both AAT and ABT) was led by the humanoid robot providing therapeutic interactions as implemented in the digital system E-BRAiN with both training standards, e.g., audio-visual instructions and feedback and individualisation algorithms, e.g., for the feedback content. For safety reasons and to step in if needed, the sessions were accompanied by the supervising staff (sitting in the background). The participants with moderate-to-severe arm paresis receiving the arm basis training cannot necessarily perform the training movements completely by themselves. Since the robot cannot provide physical assistance and serves as a social agent (therapeutic interaction), these participants need a person (“helper”) to provide physical assistance as needed for individual movements.

The scenarios, as video recorded, differed for the following two types of trainings (compare Figure 1):

A. AAT: for stroke patients with mild arm paresis, the scenario with a patient, humanoid robot, and supervising staff (three interactive agents).
B. ABT: for patients with moderate-to-severe arm paresis, the scenario with a patient, humanoid robot, helper, and supervising staff (four interactive agents).

Even though the robot is programmed to provide all therapeutic interactions necessary, there might be situations where the therapist or the helper steps in naturally and spontaneously (they are not given instructions to do so) and provides additional therapeutic interactions.

Therefore, any therapeutic interaction as performed either by a robot, therapist, or helper was documented.

The two trained raters (Ann Louise Pedersen and Philipp Deutsch) independently analysed and documented the therapeutic interactions observed in the two video-recorded sessions per participant using the instrument THER-I-ACT and its manual. THER-I-ACT measures both the occurrence/frequency and the timing of the therapeutic interactions in the thematic fields of “information provision,” “feedback,” and “bonding” with a variety of pre-defined categories in each thematic field and in addition provides a global rating of the focussed attention and engagement for both the patient and therapist (for details, see Platz et al., 2021).

### 2.4 Sample size determination

For clinical purposes, at least a moderate inter-rater reliability as indicated by an intraclass correlation coefficient (ICC) of 0.60 or higher was warranted. For testing H_0_:ICC = 0.20 (lack of reliability) *vs.* H1:ICC = 0.60 (moderate reliability) with the two independent raters and alpha = 0.05 and beta = 0.20, a sample of 27 observations would be necessary (Shoukri et al., 2004). A sample of that magnitude was planned to be recruited so that the documented ICCs of 0.6 or higher could be regarded as substantiated. Since the helper was only present and, hence, could only be observed in ABT sessions, a total of 34 observations (17 participants, AAT and ABT sessions) were included, allowing for 18 observations (ABT sessions) with a helper present.

Accordingly, the data of the first 17 participants of the E-BRAiN clinical trial were planned to be used for this study.

### 2.5 Statistical analyses

The baseline characteristics of the study population are presented using descriptive statistics (count, mean, and standard deviation).

For all THER-I-ACT measures, i.e., the frequencies and time used for the individual categories of interaction and the singular rating of the presence and engagement by the therapist (separately assessed for the robot, therapist, and helper, respectively) and of the focussed attention and engagement by the patient, the following statistics were calculated: the mean for each rater (rater 1 (R1) and rater 2 (R2)) and ICC.

The ICC is the appropriate statistic to assess the consistency of the ratings for intervals and ratio levels of the measurement (Gisev et al., 2013). In the presented research, two-way random-effects models have been used for ICC estimation, since each item was assessed by both raters. Specifically, the ICC (1, 2) according to Shrout and Fleiss (1979) had been calculated using a SAS macro written by Robert M. Hamer, Ph.D., Virginia Commonwealth University, 2-7-1991.

## 3 Results

### 3.1 Participants

The baseline characteristics of the participants are presented in Table 1.

**TABLE 1:** Study population characteristics (n = 17).

	Mean/sd	Min–max	n
	n (%)	n (%)	
Age (mean/sd; min–max)	62.4/14.3	36–81	
Sex (female; male) (n (%))	11 (65%)	6 (35%)	
Stroke type (ischaemic; ICH) (n (%))	14 (82%)	3 (18%)	
Affected brain (left; right) (n (%))	6 (35%)	11 (65%)	
Time post-stroke (weeks) (mean/sd; min–max)	86/115	3–367	
NIHSS (0–42) (mean/sd; min–max)	4.6/2.1	1–9	
Barthel index (0–100) (mean/sd; min–max)	79/18	35–100	
HADS (0–42) (mean/sd; min–max)	12.3/5.9	6–25	16^a^
FM arm^a^ (0–66) (mean/sd; min–max; n)	20.6/6.7	12–30	9
BBT^b^ (blocks/minute) (mean/sd; min–max; n)	32.5/11.6	18–44	8
NHPT^b^ (sec) (mean/sd; min–max; n)	94.9/133.2	33.0–396	7^b^
Type of training therapy (ABT^a^; AAT^b^)	8	9	

AAT, arm ability training; ABT, arm basis training; BBT, Box and Block Test; FM arm, Fugl–Meyer arm motor score; HADS, Hospital Anxiety and Depression Scale; ICH, intracerebral haemorrhage; NHPT, Nine Hole Peg Test; NIHSS, National Institute of Health Stroke Scale; NT, neglect therapy; min, minimum; max, maximum; sd, standard deviation; superscript letters (AAT^a^ and ABT^b^) indicate the different types of therapies and how they relate to both the treated syndromes and the tests used for the baseline assessment, respectively.

^a^ One participant did not want to disclose their personal emotional information.

^b^ One participant receiving AAT could not perform the NHPT during the baseline assessment.

The sample of stroke survivors recruited from as early as a few weeks to some years after a stroke included female and male participants after either right or left brain damages of the ischaemic or hemorrhagic nature with a wide age distribution and minimal to considerable disability (Barthel Index), minimal to moderate emotional distress, and a considerable range of arm motor dysfunctions (mild to severe). Hence, the sample, even though small, covered a considerable spectrum of clinical presentations that could be met after a stroke supporting a broader applicability of the study results.

### 3.2 Observed therapeutic interactions during therapy with a humanoid robot

Table 2 presents all THER-I-ACT observations made by both the independent assessors, rater 1 (R1) and rater 2 (R2), respectively. The observations from 17 participants and two sessions for each participant are presented as a group mean for all individual categories specified by THER-I-ACT and both their frequency of occurrence during a therapeutic session (count) and the time used for that type of interaction (in seconds).

**TABLE 2:** THER-I-ACT observations: The observations (mean) for individual categories by a rater (17 participants; two sessions each).

Themes and individual aspects	Mean for robot interaction				Mean for therapist				Mean for helper interaction			
	Frequency		Time used		Frequency		Time used		Frequency		Time used	
Number of sessions evaluated	34		34		34		34		18		18	
	R1	R2	R1	R2	R1	R2	R1	R2	R1	R2	R1	R2
1. Provision of information												
a. Treatment goal	2.8	2.8	130	128	<0.1	<0.1	<1	<1	0	0	0	0
b. Training specifications	0.6	0.6	79	79	0	0	0	0	0.1	0	<1	0
c. Instructions	287.5	287.2	1519	1529	8.2	7.9	34	36	29.9	30.0	57	58
2. Feedback												
a. Knowledge of performance (KP)	0	0	0	0	0.2	0.1	<1	<1	2.2	1.8	2	2
(unless corrective)												
b. KP with positive social stimuli	0	0	0	0	0.1	0.1	<1	<1	0.6	0.7	<1	1
c. KP with negative social stimuli	0	0	0	0	0	0	0	0	0	0	0	0
d. Corrective KP (cKP)	0	0	0	0	0	0	0	0	0	0	0	0
e. cKP with positive social stimuli	0	0	0	0	0	0	0	0	0	0	0	0
f. cKP with negative social stimuli	0	0	0	0	0	0	0	0	0	0	0	0
g. Knowledge of result (KR)	16.6	16.5	147	163	0.2	0.3	<1	<1	0	0	0	0
h. KR with positive social stimuli	2.2	2.2	17	17	0.2	0.2	<1	<1	0	0	0	0
i. KR with negative social stimuli	0	0	0	0	0	0	0	0	0	0	0	0
3. Motivational interactions												
a. Other than KP or KR	1.2	1.2	10	10	0.3	0.2	<1	1	0.3	0.3	<1	<1
4. Bond												
a. Showing interest in the person treated	31.4	31.6	201	207	1.7	1.9	14	13	8.6	8.8	18	17
b. Personal aspects (treating person)	0	0	0	0	0	<0.1	0	<1	0.1	0.1	<1	<1
c. Responsivity	0	0	0	0	1.4	1.6	4	4	0.5	0.5	1	1
d. Conflict solving	0	0	0	0	<0.1	<0.1	1	1	0.1	0.1	<1	<1
5. Other types of interaction	0	0	0	0	0.3	0.2	<1	<1	0.3	0.4	1	1
6. Presence (concentration) and engagement												
(treating person) (0–10)	5.0	5.0			9.0	9.5			10	9.5		
7. Focussed attention and engagement												
(patient) (0–10)	8.6	8.6			n.a.	n.a.			n.a	n.a.		
Length of the therapeutic session (minutes)	81	81										

R1 and R2, rater 1 and 2, respectively; frequency, frequency of the occurrence of a therapeutic interaction within a session (count) and the mean across observations rounded to one decimal; time used, time used for therapeutic interactions within a session (in seconds) and the mean across observations rounded to seconds (without decimals); KP, knowledge of performance; KR, knowledge of result; ^1^presence (concentration) and engagement by the supervising therapist observable and rated only for 28 sessions.

Since THER-I-ACT (Platz et al., 2021) comprehensively defines categories of therapeutic interactions, in many therapeutic situations only a subset of the possible types of therapeutic interactions can be expected.

Table 2 indicates the types, frequency, and time used for therapeutic interactions that could be observed in robot-led therapy sessions, both as provided by the humanoid robot itself (“robot interaction”) or by the supervising staff (“therapist”) or a “helper” (ABT only) spontaneously stepping in and providing the additional therapeutic interaction. As such, the table presents the general structure of the data used for inter-rater reliability analyses, descriptively, and can be verbally summarised as follows:

The observations made indicate that the humanoid robot has by far been the dominating agent providing therapeutic interactions. Its therapeutic interaction is characterised by a few longer information provision events that relate to the individual treatment goal or the applied training (i.e., AAT or ABT) in more general terms (“training specifications”) and by many short instructions given. The feedback has been given by the robot as knowledge of the results, mostly neutral (“knowledge of result”), at times associated with positive social stimuli. The work alliance supporting therapeutic interactions by the robot was not infrequently observed and fell in the category of “showing interest in person treated.”

Therapeutic interactions by the supervising staff occurred infrequently and, if so, mainly as short instructions and once or twice during a session as “showing interest in person treated.”

While again much less frequent than the robot’s therapeutic interaction, the helper spontaneously provided additional instructions and on average several times the interaction of “showing interest in person treated”, with both types of interactions, more frequently than the supervising staff.

The patients, supervising staff, and helper received high scores for the global rating of their focussed attention and engagement as perceived by the rater, while the robot received only intermediate scores.

### 3.3 Inter-rater reliability

The inter-rater reliability statistics (ICC) for individual THER-I-ACT categories as based on 34 sessions (17 participants’ first and last session with the robot and a subset of 18 sessions with a helper being present (ABT only)) are presented in Table 3.

**TABLE 3:** THER-I-ACT observations: The inter-rater reliability for individual categories (rater r = 2, participants n = 17, and sessions per participants s = 2).

Themes and individual aspects	ICC for robot interaction		ICC for therapist		ICC for helper interaction	
	**Frequency**	**Time used**	**Frequency**	**Time used**	**Frequency**	**Time used**
Number of sessions evaluated	34	34	34	34	18	18
1. Provision of information						
a. Treatment goal	1.00	1.00	1.00	1.00	n.a	n.a
b. Training specifications	1.00	1.00	n.a	n.a	1.00	1.00
c. Instructions 1.00	1.00	1.00	1.00	0.99	1.00	0.98
2. Feedback						
a. Knowledge of performance (KP)	n.a	n.a	0.96	0.96	0.99	0.96
(unless corrective)						
b. KP with positive social stimuli	n.a	n.a	1.00	1.00	0.99	0.99
c. KP with negative social stimuli	n.a	n.a	n.a	n.a	n.a	n.a
d. Corrective KP (cKP)	n.a	n.a	n.a	n.a	n.a	n.a
e. cKP with positive social stimuli	n.a	n.a	n.a	n.a	n.a	n.a
f. cKP with negative social stimuli	n.a	n.a	n.a	n.a	n.a	n.a
g. Knowledge of result (KR)	1.00	0.90	0.96	0.90	n.a	n.a
h. KR with positive social stimuli	0.99	0.99	1.00	0.96	n.a	n.a
i. KR with negative social stimuli	n.a	n.a	n.a	n.a	n.a	n.a
3. Motivational interactions						
a. Other than KP or KR	1.00	1.00	0.92	0.96	1.00	0.85
4. Bond						
a. Showing interest in the person treated	1.00	0.99	0.98	1.00	0.99	0.98
b. Personal aspects (treating person)	n.a	n.a	0.00	0.00	1.00	0.88
c. Responsivity	n.a	n.a	0.99	1.00	1.00	0.90
d. Conflict solving	n.a	n.a	1.00	0.98	1.00	0.92
5. Other types of interaction	n.a	n.a	0.79	0.34	0.92	0.98
6. Presence (concentration) and engagement						
(treating person) (0–10)	n.a^a^		0.04^b^		0.77	
7. Focussed attention and engagement						
(patient) (0–10)	0.87					
Length of the therapeutic session (minutes)	1.00					

Aside from the number of sessions (= count), all the other statistics provided are ICCs, for the consistency of measurements between the two independent raters (rater data presented in Table 2). ICC, intraclass correlation coefficient; frequency, frequency of occurrence of therapeutic interactions within a session (count); time used, time used for therapeutic interactions within a session (in seconds); KP, knowledge of performance; KR, knowledge of result; n.a., not applicable or the type of interaction not observed.

^a^ Presence (concentration) and engagement by the robot invariably rated as “5”.

^b^ Presence (concentration) and engagement by the supervising therapist rated only for 28 sessions.

For all categories of therapeutic interaction by the humanoid robot, the ICC was ≥0.90 for both the frequency of occurrence and time used for the type of interaction indicating a high degree of reliability.

Even though rather infrequently observed, therapeutic interactions by the supervising staff (“therapist”) could mostly be documented reliably (ICC ≥0.90) with two exceptions, i.e., introducing own personal aspects and (any) “other type of interaction”; both of them occurred only exceptionally.

The therapeutic interaction by a helper (ABT sessions only), while somewhat more frequently observed than the interaction by the supervising staff, yet much less than the interaction by the robot, could nevertheless be reliably documented (ICC ≥0.85).

The ICC was similarly high for an overall subjective rating of the concentration and engagement of patients (ICC 0.87), somewhat less, but still substantial for the helper (ICC 0.77), but not for the rating “presence and engagement” of the supervising staff sitting in the background (ICC 0.00).

## 4 Discussion

With the number of people living with the aftermath of stroke being on the rise globally (Nguyen et al., 2019) and the intensive individualised rehabilitative training having the potential to reduce stroke-related disabilities (Joy and Carmichael, 2021), one way that had been entertained to support prolonged rehabilitation is the use of digital therapeutic systems based on the HR technology.

The acceptance and use of the HR technology will likely be influenced by the enjoyment and ease of use experienced by its users and their trust in the HR application (Jung et al., 2021). The factors related to the robot itself, specifically, its functional performance, seem to have the greatest impact on trust (Hancock et al., 2011; Koren et al., 2022). Such functional performance is related to the specificity of the training provided by an HR application, its potential to adapt to the individual necessities, and any training progress, as well as its therapeutic interaction.

Indeed, research on the use of HRs for training-based rehabilitation has acknowledged the relevance of and implemented therapeutic interactions as verbal and non-verbal (e.g., demonstration) instructions and performance-based feedback (Martin et al., 2020; Blanchard et al., 2022; Koren et al., 2022).

The research on human–robot interactions, thus far, has focussed on the users’ perspective and assessed enjoyment, ease of use, and trust from the users’ perspective (Jung et al., 2021). The functional performance of an HR, however, is defined on the robot’s side with therapeutic interactions being an integral part of it. Given its prominent role, a standardised assessment of an HR’s therapeutic interaction would be equally warranted.

In this research, the instrument THER-I-ACT that had been developed and validated for the situation when a human therapist interacts with patients therapeutically (Platz et al., 2021) has now been assessed when applied to document the therapeutic interaction performed by an HR. Here, it could be demonstrated that the therapeutic interaction of an HR leading neurorehabilitation training sessions as a social agent providing information, giving instructions and feedback, and taking interest in a person during training could equally, comprehensively, and reliably be assessed with THER-I-ACT. The ICC was ≥0.90 for both the frequency of occurrence and time used for all types of the robot’s therapeutic interactions. This indicates a very high degree of reliability for the documentation of these highly detailed categories of therapeutic interactions by two independent trained assessors.

In addition, this research extended the scope of the assessment of the therapeutic interaction using THER-I-ACT for situations where not only a therapist and a patient are present but also when a humanoid robot is the primary therapeutic coach, with the supervising staff being present at the same time and, at times, with the presence of a human “helper” who provides physical assistance as needed (ABT). It could be shown for these scenarios that not only the therapeutic interaction by the humanoid robot but also the additional (while much less frequent) spontaneously occurring therapeutic interaction both by the supervising staff and a helper could reliably be documented. The exceptions were the interactions that hardly ever occurred.

The limitations of the research that are noteworthy are the limited sample and the types of therapies assessed. While the sample of stroke survivors included showed a relevant variability of sociodemographic and clinical characteristics and while the two types of therapies (i.e., AAT and ABT) have different characteristics and associated therapeutic interactions, it remains a possibility that the high degree of the inter-rater reliability observed might not equally apply to all therapeutic situations (e.g., different patients groups, other types of therapies, and therapeutic interaction categories that were not observed in this research, e.g., the corrective knowledge of the performance). In addition, the high degree of the inter-rater reliability was documented when the raters had been well trained to use the instrument. It is, however, conceivable that reaching the competence to assess therapeutic interactions of an HR necessitates less training for a rater compared to that of the assessment of a human agent’s therapeutic interaction. The algorithmic setup of an HR’s interaction makes it more standardised (even when individualised) compared to the spontaneous human communication that can have a complex structure and a high degree of variability.

In conclusion, the research data presented support the notion that therapeutic interactions can reliably be assessed with the instrument THER-I-ACT, not only in the traditional patient–therapist setting, as shown previously (Platz et al., 2021), but also in a digital setting with a humanoid robot as the therapeutic agent. As such, it offers the possibility to perform a video-based assessment of an HR’s therapeutic interaction as one aspect of its functional performance. Furthermore, THER-I-ACT can reliably be used to document the therapeutic interaction for scenarios where more than one therapeutic agents are present, e.g., when both a humanoid robot and human agents provide therapeutic interactions. As such, the data support the use of THER-I-ACT for these situations and extend the instrument’s validated application context.

## Data Availability

The datasets presented in this article are not readily available because the data can only be used for the purpose and by the persons agreed to, with informed written consent by participants. Requests to access the datasets should be directed to thomas.platz@uni-greifswald.de.
